# Education Burnout and Engagement in Occupational Therapy Undergraduate Students and Its Associated Factors

**DOI:** 10.3389/fpsyg.2019.02889

**Published:** 2019-12-20

**Authors:** Francisco Manuel Morales-Rodríguez, José M. Pérez-Mármol, Ted Brown

**Affiliations:** ^1^Department of Educational and Developmental Psychology, Faculty of Psychology, University of Granada, Granada, Spain; ^2^Department of Physiotherapy, Faculty of Health Sciences, University of Granada, Granada, Spain; ^3^Department of Occupational Therapy, Faculty of Medicine, Nursing and Health Sciences, School of Primary and Allied Health Care, Monash University, Peninsula Campus, Frankston, VIC, Australia

**Keywords:** burnout, engagement, undergraduate students, education, academic

## Abstract

**Introduction:**

Burnout syndrome has been characterized as a process of chronic responses to occupational stress in certain employee groups. However, this phenomenon has also been reported in other participant groups including university students. The Maslach Burnout Inventory-Student Survey (MBI-SS), composed of the Exhaustion, Cynicism and Efficacy subscales, was used to evaluate burnout in this sample group while the Utrecht Work Engagement Scale (UWES) was used to gather data related to engagement, a positive psychology construct composed of the three factors, namely vigor, dedication, and absorption. To date, no studies considered these factors in relation to occupational therapy students. This begs the question, is there a relationship between occupational therapy students’ self-reported levels of burnout and engagement?

**Objectives:**

The study objectives are to (1) ascertain the self-reported levels of burnout and engagement in a sample of Australian Occupational undergraduate therapy students, and (2) analyze the sociodemographic, occupational and academic characteristic associated with these levels.

**Methods:**

Participants were 225 Australian undergraduate occupational therapy students from Monash University completed the MBI-SS and the UWES for students. Descriptive, bivariate and multiple linear regression analyses were performed.

**Results:**

Regarding MBI-SS burnout dimensions, exhaustion was associated with age, year level of enrolment and hours of direct time spent working on occupational therapy studies, explaining 15% of its variance. Cynicism and efficacy were associated with age, year level of enrolment and hours of indirect time, accounting for 16% of its variance. For the UWES engagement dimensions, year level of enrolment and hours of indirect time spent working on occupational therapy studies were significant predictors of vigor, explaining 27% its variance while while age, gender, year level of enrolment, hours of indirect time spent working on occupational therapy studies, and hours spend per week engaged in self-care activities accounted for 23% of the variance of dedication. Finally, age, year level of enrolment, and hours of indirect time spent working on occupational therapy studies explained 27% of the variance of absorption.

**Conclusion:**

The results indicate that a number of demographic and academic study variables are significantly associated with burnout syndrome and education engagement reported by undergraduate occupational therapy students.

## Introduction

Burnout syndrome has been described as a process of chronic responses to occupational stress in certain groups of workers. The dimension of *burnout* has been classically framed in the three-dimensional theoretical model of Maslach and Jackson (1981), which is in line with other more recent models such as that of Schaufeli et al. (2002a); Salanova et al. (2005), Caballero and Breso (2015), or Hederich-Martínez and Caballero-Domínguez (2016). Burnout syndrome in students has been evaluated by the Maslach Burnout Inventory-Student Survey (MBI-SS) that is made up of three subscales: *exhaustion, cynicism*, and *efficacy* (Schaufeli et al., 2002a). However, this phenomenon has also been reported in other participant groups including university students. Therefore, the study of burnout has been extended to pre-occupational samples, referring to it as *academic burnout*.

In the academic field, the models derived from the “social exchange” theory (Cropanzano et al., 2017; Hernández-Oñativia, 2018) are developed based on the approach of a continuous situation of imbalance between the student’s personal involvement and the perceived social rewards. Specifically, within this theory, burnout may be understood under the “job demands-resources” model of Bakker and Demerouti (2007) and the “Conservational of Resources” model (Robins et al., 2015) which, in turn, results from the combination of demand/control and effort/reward models (Rodríguez-Muñoz et al., 2006; Hernández-Oñativia, 2018). Empirical results have shown that students may also become burnt-out for their studies, showing high levels of exhaustion, cynicism, and lack of efficacy regarding their academic activities (Salanova et al., 2000; Agust et al., 2001; Martínez et al., 2002; Schaufeli et al., 2002a).

On the other hand, *engagement* as a factor has gain attention in the educational literature due to the focus in recent years toward positive psychology, which centers on optimal development and positive aspects and not on dysfunction (Seligman and Csikszentmihalyi, 2000; Schaufeli and Bakker, 2004a, b; Zabuska et al., 2018). According several authors, engagement should be viewed as an opposite concept in relation to burnout (Poulsen et al., 2014; Robins et al., 2015; Martos et al., 2018). More than a specific state, engagement refers to a more stable affective-cognitive state that is not focused on a particular object, event or situation (Schaufeli and Bakker, 2004b). Engagement involves three factors: (i) *vigor*, defined as high energy levels and mental flexibility as well as the ability to spend effort in one’s studies; (ii) *dedication*, referred to as a sense of importance, enthusiasm, inspiration and self-esteem; and (iii) *absorption*, defined as being fully concentrated and happily absorbed in one’s study, by means of which time passes rapidly (Schaufeli et al., 2002a).

As Schaufeli and Bakker (2004b) suggested in an integrative effort of “job demands-resources” model, when studying engagement in university students, researchers should also focus their attention on the influence of variables of the work environment and personal resources. The study of these variables could provide educators with a clearer picture of the related factors to students’ engagement in university environments. Although the research on engagement is less extensive, given the relative novelty of the concept, previous results have shown the positive influence of engagement in personal and social functioning in various contexts, such as academic performance (Schaufeli et al., 2002b; Salanova et al., 2003).

A link has been established between academic burnout and the use of unproductive or ineffective strategies for managing stress (Schaufeli and Salanova, 2007) and poor academic performance in students enrolled in a number of health care fields (including medicine, nursing, psychology and physiotherapy) (Caballero and Breso, 2015). By contrast, engagement has been linked to good academic performance (Caballero and Breso, 2015; Gómez et al., 2015). Authors such as Fernández-Martínez et al. (2017) have found that promoting positive engagement in university studies can improve student support and communication networks. Kroska et al. (2017) found that an increased amount of avoidant coping strategies and lower levels of engagement/commitment were associated with elevated levels of depressive symptoms and burnout in medical students.

Other research has demonstrated the link between evaluated levels of burnout and engagement in the educational context; however, these have been conducted in the areas of educational sciences, labor relations, and human resources (Vizoso Gómez and Arias Gundín, 2016). In turn, other authors found in a sample involving 18 different academic degree areas of study that the students with the highest academic performance exhibited less burnout and higher levels of engagement (Salanova et al., 2005). Also, academic burnout syndrome was negatively associated with happiness in the face of studies (Salanova et al., 2005) and positively with an increased level of anxiety symptoms (Caballero and Breso, 2015) and with lower academic performance outcomes (Martínez Martínez and Pinto Marques, 2005; Caballero and Breso, 2015; Gómez et al., 2015).

Not so much is known about the influence of demographic characteristics of university students have on their burnout and engagement. In terms of age, Chiddarwar and Singh (2016) found no association of burnout with age. Robins et al. (2015) found that the average scores were higher in engagement and lower in exhaustion/burnout among students over 30 years of age compared with those 29 years of age or younger. Regarding gender differences in burnout, although most literature has produced inconsistent (Maslach et al., 2001), Purvanova and Muros (2010) reported in a meta-analysis that women tend to feel more exhausted and men report higher depersonalization. Other findings reported no differences in burnout levels according to gender (Backoviæ et al., 2012; Robins et al., 2015; Peralta-Ayala and Moya, 2017; Martos et al., 2018). The study conducted by Zabuska et al. (2018) also informed no differences between men and women in their self-reported engagement. Nevertheless, these authors showed that women displayed higher levels of global burnout and emotional/physical exhaustion, while men reported lower levels of sense of accomplishment (Zabuska et al., 2018). In addition, according to Römer (2016), burnout was significantly more prevalent in females (75%) than males (25%). These findings may be probably related to gender stereotyping within the education context. Other authors discuss that the development of burnout as a response to chronic stress partly explained by sex-dependent biological mechanisms underlying responses to stressors (Goldstein et al., 2010). Additionally, the process of socialization plays a key role, encouraging social dependence in women, and independence associated with the sense of competence in men (Dedovic et al., 2009). The study of Taka et al. (2016) suggests that women may be more psychologically vulnerable to the organizational climate for gender inequality than their male counterparts.

With regard to the academic year, Gómez et al. (2015) found high levels of engagement with their studies and low levels of burnout in first-year medical students. Fernández-Martínez et al. (2017) also show that first year students scored higher in engagement and resilience factors in comparison to those of in the second and third years. On the contrary, Parada Contreras and Pérez Villalobos (2014) showed that being of a female student in their final year of a last course were contributing factors to higher academic engagement scores in dentistry students, that is, they were more committed in general and involved in their studies. Significant statistical differences have also been found with regard to emotional exhaustion according to the course, with higher levels observed during the third and second years, respectively. In the second course, the lowest levels of depersonalization were found, which according to the authors could be explained by the absence of contact with patients through practical rotations (Kroska et al., 2017). Likewise, Robins et al. (2015) found that students in their final year of a course scored higher in burnout and engagement, in comparison with those prior to their final year. The fact that there is a link between the academic course and engagement may be due to the important role in these courses of developing clinical practice skills which allow more direct contact with the professional realities of discipline (Gupta et al., 2012). Regarding other academic characteristics, Taka et al. (2016) found that “work hours” was a significant factor directly associated with burnout scores, and Shah and Cheng (2019) found that online students are less engaged in learning and, therefore, efforts were needed to improve their sense of belonging to the university.

In the area of occupational therapy, there is research that has examined the influence of various factors on burnout in occupational therapy professionals, such as academic stress (Tyrrell and Smith, 1996; Yang et al., 2006). Evidence has been found that these constructs are associated with mental health and well-being in already graduated occupational therapists (Bassett and Lloyd, 2001; Zeman and Harvison, 2017). Poulsen et al. (2014) investigated the relationship between sociodemographic and labor characteristics in a sample of occupational therapy staff. A previous study also examined the levels of burnout of occupational therapists by applying the Oldenburg Burnout Inventory and it was found that high levels of emotional exhaustion and cynicism with consequences such as high levels of job dissatisfaction, low organizational commitment, and higher rates of work absenteeism (Gupta et al., 2012). As far as we know, no studies have considered any factors associated with burnout syndrome and engagement involving occupational therapy students. Therefore, until now, the effect that factors such as sex, age and time spent on extracurricular activities, among other sociodemographic characteristics, have on burnout and engagement is still unclear.

More research is required in the educational context, especially among occupational therapy students, due to the impact generated by these constructs at an educational level and on the health and well-being of students with this group. In that way, this information could: (1) generate a greater awareness in educators of the real emotional state of their students, (2) make it possible to design psychoeducational interventions which could engage students in the most effective or productive coping strategies, (3) allow the approach of the factors that are related or that modulate these constructs, and (4) contribute to a higher quality of life and well-being in university students. Therefore, the main objectives of the present study are: (1) to analyze the levels of burnout and engagement in a sample of Australian occupational therapy undergraduate students, and (2) to analyze the sociodemographic, occupational and academic characteristics associated with these levels. We hypothesize that there is a relationship between burnout and engagement, and sociodemographic, occupational and academic characteristics.

## Materials and Methods

### Design

For this study, a cross sectional non-experimental research design was selected involving convenience sampling of student participants. Participants were students recruited from the health science (occupational therapy) courses at the Monash University and were asked to complete a series of self-report scales with a Likert response format. The data were analyzed using correlation and multiple regression statistics.

### Participants

The questionnaires were completed by 225 Australian occupational therapy undergraduate students. The students were enrolled in the first, third and fourth year the bachelor of occupational therapy (Honors) course that is offered at Monash University, Peninsula Campus, Frankston, VIC, Australia. Second year students were not able to be recruited as part of the sample since they were not accessible due to the scheduling of their tutorials and lectures. The rate response was of 100%.

Monash University Human Research Ethics Committee approval was obtained on the 9 April 2015 (project number: 2015-6069-5898). This study met the requirements of the National Statement on Ethical Conduct in Human Research and was conducted in accordance with the Declaration of Helsinki 2013.

### Instruments

A demographic, occupational and academic questionnaire was used to collect relevant information about the participant group such as age, gender, year level of enrolment, enrolment status, entrance to education program directly from high school, previous educational experience, direct occupational therapy class time attended per week (hours), indirect time spent working on occupational therapy studies per week (hours), hours per week students spend working at a paid job while completing occupational therapy studies, and hours occupational therapy students spend per week engaged in self-care activities (Appendix 1). The levels of burnout and engagement dimensions reported by students were obtained using the MBI-SS and UWES scales.

The MBI-SS – MBI-SS consists of 15 items that are allocated to one of three subscales: Exhaustion with five items, Cynicism with four items, and Professional/Academic Efficacy with six items. The MBI-SS uses a seven-point frequency rating scale that ranges from 0 (never) to 6 (every day) for participants to rate each item. High scores on the Exhaustion and Cynicism subscales and low scores on the Professional/Academic Efficacy scale are interpreted as indicating the presence of burnout in respondents. The Rasch Measurement Model (RMM) analysis of the three MBI-SS individual subscales for the current sample exhibited adequate levels of structural validity in relation to dimensionality and differential item functioning, i.e., occupational therapy students male and female responded in a similar manner to individual scale items measuring the same underlying construct. Results supported the unidimensionality and scalability (or interval level scaling means that the units of measure reflect equal quantities across the range of the construct) of the three individual MBI-SS. The item and person reliability indices were >0.80 and >0.77, respectively, for each subscale (Pérez-Mármol and Brown, 2018). Additionally, for the current sample of the study, this instrument exhibited a satisfactory reliability with a Cronbach alpha of 0.90 for exhaustion, 0.88 for cynicism, and 0.85 for academic efficacy.

The Utrecht Work Engagement Scale (UWES) for students (Schaufeli et al., 2002a) has been widely validated in Australia, and in many other countries. Six items measure Vigor, five items measure Dedication, and the remaining six items measure Absorption. Confirmatory factor analyses have repeatedly demonstrated an adequate three-factor structure fit, with satisfactory internal consistencies of the three sub- scales (Schaufeli et al., 2002a, b). Items are scored on a scale of 0 (never) to 6 (always) to determine subscale scores for Vigor, Dedication, and Absorption, which are combined to produce a total score for Work Engagement. Internal reliability is good and factor structure demonstrates stability across industries and countries (Schaufeli et al., 2002a). The scale exhibited satisfactory reliability for the current sample of the study with a Cronbach alpha of 0.83 for vigor, 0.89 for dedication, and 0.87 for absorption.

### Procedures

Undergraduate students were approached at the conclusion of a lecture during Semester One 2015 by a non-teaching member of staff and were asked to complete a hard copy of the questionnaire containing the UWES and MBI-SS. Consent was inferred if students volunteered to complete and return the questionnaire. It took participants on average 10 min to complete the self-report questionnaire. Students placed the completed questionnaires in the back of the lecture theater as they exited.

### Statistical Analysis

Statistical analysis was performed using SPSS software, version 21.0. First, descriptive analyses were calculated and the normal distribution of the variables was confirmed with the Kolmogorov Smirnov test. To determine the association between demographic academic variables, levels of burnout and engagement, Pearson Correlation Coefficients were used. A scatterplot was used to check the violation of the assumptions of linearity and homoscedasticity and decide if we had to use parametric or non-parametric statistics. To evaluate the influence of sociodemographic, occupational and academic variables on MBI-SS and UWES scale scores, a multiple linear regression analysis was performed with dimensions of burnout and engagement as the dependent variables, and the rest of characteristics as independent variables. We used a stepwise method by entering all variables into the regression analyses (León and Montero, 2015). The stepwise regression model made an evaluation of the collinearity to decide which independent variables were included in the final model. A *p*-value less than 0.05 was considered as a statistically significant value in all cases.

## Results

### Sample Description

The total sample consisted of 200 female (88.9%) and 25 male (11.1%) students with an average age of 21.64 years (*SD* = 4.11). Other participant group demographic variables are reported in Table 1. Descriptive data of dimensions of burnout and engagement for the total sample is shown in Table 2.

**TABLE 1:** Sociodemographic and occupational characteristics of the undergraduate occupational therapy student sample (*N* = 225).

**Sociodemographic and occupational variables**	**Frequency**	**Percentage (%)**
**Year level of enrolment**		
First year	97	43.10
Third year	71	31.60
Fourth year	57	25.30
**Enrolment status**		
Full-time	215	95.60
Part-time	10	4.40
**Entrance to education program directly from high school?**		
Yes	112	49.80
No	113	50.20
**Previous educational experience?**		
Yes	111	49.3
No	114	50.7

	**Range (minimum–maximum)**	**Mean (*SD*)**

Direct occupational therapy class time attended per week (hours)	2–65	15.85 (6.66)
Indirect time spent working on occupational therapy studies per week (hours)	0–45	15.19 (8.03)
Hours per week students spend working at a paid job while completing occupational therapy studies	0–35	10.90 (8.05)
Hours occupational therapy students spend per week engaged in self-care activities	1–100	16.32 (16.31)

*SD, standard deviation.*

**TABLE 2:** Descriptive data of dimensions of burnout and engagement for the total sample of occupational therapy university students.

	***N***	**Mean**	***SD***	**Range**	**Q1**	**Q2**	**Q3**
**MBI-SS dimensions**							
Exhaustion	224	17.58	6.36	0.00–30.00	13.25	18.00	22.00
Cynicism	222	5.77	5.05	0.00–21.00	1.00	4.50	9.00
Efficacy	223	26.55	5.33	6.00–36.00	23.00	27.00	31.00
**UWES dimensions**							
Vigor	222	16.16	5.36	0.00–29.00	12.00	16.00	21.00
Dedication	223	22.05	4.93	7.00–30.00	19.00	23.00	25.00
Absorption	218	13.14	5.56	0.00–24.00	9.00	14.00	17.25

*SD, standard deviation; Q1, Q2, and Q3, quartile scores; MBI-SS, Maslach Burnout Inventory-Student Survey; UWES, Utrecht Work Engagement Scale.*

### Descriptive Results for MBI-SS Burnout Dimensions and UWES Engagement Dimensions

Descriptive statistics for the MBI-SS and UWES engagement scores are presented in Table 2.

### Relationship Between MBI-SS Burnout Dimensions on UWES Engagement Dimensions

Bivariate Pearson correlation between burnout dimensions and engagement dimensions in occupational therapy university students (*N* = 225) are shown in Table 3. Bivariate correlation analyses showed an inverse association between exhaustion and vigor (*p* < 0.001), an inverse association between Cynicism and Dedication (*p* < 0.001), and a direct association between Efficacy and Dedication (*p* < 0.001).

**TABLE 3:** Bivariate Pearson’ correlation between burnout dimensions and engagement dimensions in occupational therapy university students (*N* = 225).

**MBI-SS dimensions**	**UWES dimensions**	**Pearson *r***
Exhaustion	Vigor	−0.329^∗∗^
	Dedication	−0.192^∗∗^
	Absorption	−0.212^∗∗^
Cynicism	Vigor	−0.482^∗∗^
	Dedication	−0.498^∗∗^
	Absorption	−0.338^∗∗^
Efficacy	Vigor	0.558^∗∗^
	Dedication	0.709^∗∗^
	Absorption	0.487^∗∗^

*^∗∗^*p* < 0.001; MBI-SS, Maslach Burnout Inventory-Student Survey; UWES, Utrecht Work Engagement Scale.*

### Association Between Burnout and Engagement, and Sociodemographic, Occupational and Academic Characteristics

Bivariate correlation analyses showed a direct and inverse association between burnout and engagement, and all sociodemographic, occupational and academic characteristics, except for gender and enrolment status. These findings are shown in Table 4.

**TABLE 4:** Bivariate Pearson’ correlation analysis between burnout and engagement, and sociodemographic and occupational characteristics.

**Variables**	**MBI-SS dimensions**			**UWES dimensions**		
		
	**Exhaustion**	**Cynicism**	**Efficacy**	**Vigor**	**Dedication**	**Absorption**
Age	–0.174^∗∗^	–0.119	0.095	0.023	0.102	0.125
Gender	–0.097	0.059	–0.090	–0.077	–0.090	–0.075
Year level of enrolment	0.239^∗∗^	0.304^∗∗^	–0.270	–0.471^∗∗^	–0.353^∗∗^	–0.367^∗∗^
Enrolment status	–0.081	0.018	0.047	0.010	0.033	0.042
Entrance to education program directly from high school?	–0.081	–0.096	0.145^∗^	0.015	0.132^∗^	0.127
Previous educational experience?	0.115	0.139^∗^	–0.085	–0.032	0.118	–0.088
Hours of direct time	0.283^∗∗^	0.115	0.009	–0.066	–0.055	–0.011
Hours of indirect time	–0.043	–0.184^∗∗^	0.261^∗∗^	0.237^∗∗^	0.231^∗∗^	0.333^∗∗^
Hours per week working at a paid job	0.125	0.049	–0.053	−0.136^∗^	–0.043	–0.096
Self-care activities (hours/per week)	–0.043	0.097	−0.136^∗^	–0.100	−0.152^∗^	–0.046

*^∗^*p* < 0.05; ^∗∗^*p* < 0.001; MBI-SS, Maslach Burnout Inventory-Student Survey; UWES, Utrecht Work Engagement Scale.*

Regarding burnout dimensions, results after multiple regression analyses have shown that exhaustion was significantly associated with age (*p* = 0.014), year level of enrolment (*p* < 0.001), and hours of direct time spent working on occupational therapy studies (*p* < 0.001), explaining 15% its variance. Cynicism and efficacy were associated with age (*p* < 0.05), year level of enrolment (*p* < 0.001) and hours of indirect study time (*p* < 0.001), explaining 16% its variance. These results are shown in Table 5.

**TABLE 5:** Regression model of sociodemographic and occupational characteristics predictive associated factors to emotional exhaustion, cynicism, and efficacy in university students.

**Independent variables**	***B***	**95% CI**		**β**	***SE***	***p*-value**
		**Lower bound**	**Upper bound**			
**Emotional exhaustion (*r*^2^ = 0.150)**						
Age	−0.283	−0.508	−0.058	−0.165	0.114	0.014
Year level of enrolment	1.320	0.659	1.980	0.265	0.335	<0.001
Hours of direct time	0.231	0.110	0.352	0.244	0.061	<0.001
**Cynicism (*r*^2^ = 0.164)**						
Age	−0.228	−0.409	−0.046	−0.165	0.092	0.014
Year level of enrolment	1.492	0.961	2.002	0.372	0.269	<0.001
Hours of indirect time	−0.083	−0.163	−0.002	−0.131	0.041	<0.001
**Efficacy (*r*^2^ = 0.156)**						
Age	0.213	0.019	0.407	0.145	0.098	0.032
Year level of enrolment	−1.216	−1.785	−0.648	−0.285	0.288	<0.001
Hours of indirect time	0.158	0.072	0.243	0.237	0.043	<0.001

**r*^2^, regression coefficient of determination; *B*, regression coefficient; CI, confidence interval; β, adjusted coefficient from multiple linear regression analysis; *SE*, coefficient standard error.*

In relation to engagement dimensions, year level of enrolment (*p* < 0.001) and hours of indirect time spent working on occupational therapy studies (*p* < 0.001) were significant predictors of vigor, explaining 27% its variance, while age (*p* = 0.023), gender (*p* = 0.034), year level of enrolment (*p* < 0.001), hours of indirect time spent working on occupational therapy studies (*p* = 0.001), and hours spend per week engaged in self-care activities (*p* = 0.016) accounted for 23% of the variance of dedication. On the other hand, age (*p* = 0.001), year level of enrolment (*p* < 0.001), and hours of indirect time spent working on occupational therapy studies (*p* < 0.001) explained 27% of the variance of absorption. These findings are shown in Table 6.

**TABLE 6:** Regression model of sociodemographic and occupational characteristics predictive associated factors to vigor, dedication, and absorption in university students.

**Independent variables**	***B***	**95% CI**		**β**	***SE***	***p*-value**
		**Lower bound**	**Upper bound**			
**Vigor (*r*^2^ = 0.271)**						
Year level of enrolment	−1.873	−2.367	−1.379	−0.448	0.251	<0.001
Hours of indirect time	0.144	0.067	0.221	0.221	0.039	<0.001
**Dedication (*r*^2^ = 0.228)**						
Age	0.199	0.28	0.371	0.148	0.087	0.023
Gender	−2.134	−4.099	−0.168	−0.132	0.997	0.034
Year level of enrolment	−1.417	−1.919	−0.914	−0.360	0.255	<0.001
Hours of indirect time	0.124	0.049	0.199	0.203	0.038	0.001
Self-care activities (hours/per week)	−0.047	−0.085	−0.009	−0.151	0.019	0.016
**Absorption (*r*^2^ = 0.274)**						
Age	0.308	0.120	0.496	0.204	0.095	0.001
Year level of enrolment	−1.767	−2.318	−1.216	0.287	0.042	<0.001
Hours of indirect time	0.197	0.115	0.280	0.204	0.095	<0.001

**r*^2^, regression coefficient of determination; *B*, regression coefficient; CI, confidence interval; β, adjusted coefficient from multiple linear regression analysis; SE, coefficient standard error.*

## Discussion

The first aim of the study was to analyze the levels of burnout and engagement in a sample of Australian occupational therapy undergraduate students. The recruited sample exhibit low-moderate exhaustion, low cynicism and high efficacy levels; and moderate levels of all engagement dimensions. The results showed that burnout dimensions were inversely related to engagement dimensions in the sample examined. The second aim was to investigate what demographic, occupational and academic factors of university occupational therapy students were associated with and predictive of their burnout levels and educational engagement. Regarding the factors linked to burnout dimensions, results have shown that exhaustion was correlated with age, year level of enrolment and hours directly spent working on occupational therapy studies; cynicism and efficacy were related to age, “which level or year the student is enrolled in,” and hours of indirect time. With regards to the engagement dimensions, vigor was associated with “which level or year the student is enrolled in,” and hours of indirect time spent working on occupational therapy studies; dedication was related to age, gender, “which level or year the student is enrolled in,” hours of indirect time spent working on occupational therapy studies, and hours spent per week engaged in self-care activities; and absorption with age, year level of enrolment, and hours of indirect time spent working on occupational therapy studies.

Burnout has been classically framed in the three-dimensional theoretical model of Maslach and Jackson (1981) as being the result of chronic academic-related stress, versus engagement as a positive construct in the academic environment. This theory is in line with other more recent models (Schaufeli et al., 2002a; Salanova et al., 2005; Caballero and Breso, 2015; Hederich-Martínez and Caballero-Domínguez, 2016). From the models derived from the “social exchange” theory (Cropanzano et al., 2017; Hernández-Oñativia, 2018), burnout and engagement may be understood under the “job demands-resources” model of Bakker and Demerouti (2007) and the “conservational of resources” model (Robins et al., 2015) which, in turn, results from the combination of demand/control and effort/reward models (Rodríguez-Muñoz et al., 2006; Hernández-Oñativia, 2018). Present results are consistent with these theoretical models since burnout dimensions were inversely related to engagement dimensions (and vice versa) in the sample examined. The self-reported levels of burnout and engagement in this population ranged between low and moderate. Moreover, the current findings show that several demographic, occupational, and academic factors of university students are associated with and are predictive of their academic burnout and engagement.

Regarding the levels of burnout and engagement, a cross-national study with students from Australia, Poland, and the United Kingdom (Zabuska, 2017) showed low levels of burnout (although a ten percent of students were at risk), and moderate degrees of engagement. In a similar study, Zabuska et al. (2018) reported low to moderate self-reported levels of burnout, where Australian and British students displayed higher levels of burnout than Polish students did. Eleven percent of all participants were at risk of academic burnout. This study also revealed differences for levels of exhaustion between Australian and Polish respondents, being higher in Australia; and there were differences for reduced sense of accomplishment, being lower for Australian than Polish and British student respondents. Inversely, these students showed moderate to high self-reported levels of engagement (Zabuska et al., 2018). Backoviæ et al. (2012) studied burnout in a sample of medical students and found high levels of depersonalization and emotional exhaustion in this group. Bernhard (2007, 2010) observed high exhaustion, and moderate depersonalization and personal accomplishment in American university students. Other results evidence higher levels of burnout in countries such as North America in comparison with regions in Europe (van Horn et al., 1997). These findings are in line with previous investigations that reported different findings on burnout and engagement levels even among European countries (Taipale et al., 2011). Therefore, results on burnout and engagement levels seem to be heterogeneous among countries, being crucial conducting specific studies in each one of them.

Regarding the levels of burnout and engagement, as in the present study, other previous research (Zabuska, 2017) showed an association between levels of burnout and engagement in Australian students. An inverse relationship between burnout and engagement levels has been previously reported in studies of health professional students (Poulsen et al., 2014; Robins et al., 2015; Martos et al., 2018), and in already qualified occupational therapists (Poulsen et al., 2014). Burnout in occupational therapists group has been associated with low satisfaction levels due to factors such as low income, having less than 10 years of professional experience, not having children, presence of work overload, low assertiveness or difficulty in saying “no,” lack of psychologically switching off from work outside of working hours and fewer predisposition to a sense of humor. On the other hand, engagement has been linked to having or not having children, having completed a postgraduate qualification, a greater predisposition to a sense of humor, a working week exceeding 40 h, satisfaction due to high income and a strong psychological connection with work (Poulsen et al., 2014). In line with this, the impact of possible protective or risk factors on burnout and engagement in occupational therapy students could generate a better understanding of the influence of them on their educational experience.

Regarding gender differences in burnout, our findings are partially in line with those of Backoviæ et al. (2012); Robins et al. (2015), Peralta-Ayala and Moya (2017); Martos et al. (2018), and Zabuska et al. (2018), who reported no differences in burnout levels according to gender. Regarding academic characteristics, a study by Zabuska et al. (2018) evaluated if full-time versus part-time enrolment status of Polish students had an influence on burnout and engagement; however, the only significant difference was found for absorption, reporting higher levels in students enrolled in part-time versus full-time studies. A study found that online students are less engaged in learning and, therefore, efforts were needed to improve their sense of belonging to the university (Shah and Cheng, 2019). Another investigation reported that burnout was directly associated to the number of hours worked. That is, for the outcome of student-related burnout, “work hours” was a significant factors directly associated with burnout scores (Taka et al., 2016). On the other hand, Robins et al. (2015) found no statistically significant differences in terms of academic discipline. We found that the average number of hours of direct and indirect time spent working on occupational therapy studies and the average number of hours occupational therapy students spend per week engaged in self-care activities were significantly associated with burnout and engagement. Therefore, the academic and occupational variables appear to have an influence on these psychological dimensions in university settings.

The findings help to inform the understanding of how sociodemographic, academic and occupational characteristics may be related to the development of students’ academic burnout and engagement in higher education contexts. In this sense, the organizational, personal and social obstacles faced by university students in this environment could be managed in a better manner, generating a greater awareness in educators and students about the real emotional state of their students. The data obtained could be useful for the promotion of wellbeing, positive personal resources, a decrease in the levels of academic mental health stress and better performance by occupational therapy undergraduate students. Furthermore, this information is relevant to prevent exhaustion and make recommendations regarding the issue of engagement. The students could find in their surroundings more aids and fewer obstacles, probably contributing to improvements in their future performance (Salanova et al., 2010). This view is reinforced by other studies that find that academic burnout can be predicted by strong feelings of inefficiency (Bresó et al., 2011) and a lower level of emotional intelligence that otherwise could be a protection against it (Caballero and Breso, 2015). Schaufeli and Salanova (2007) found in a sample of Spanish university students that believing that oneself was inefficient negatively correlated with the vigor, dedication and absorption factors in the engagement construct.

### Limitations of the Study and Future Research

Some limitations should be considered in the present study. First, the study employed a cross-sectional design and this design does not allow establishing causal relationships between the investigated variables. In the future, it is necessary to perform studies with a longitudinal design to evaluate the directionality of the relationships (cause–effect relationship), i.e., to know the variables that have an influence on the levels of burnout and engagement. These findings will make possible to have valuable information for developing psychoeducational interventions for the prevention of burnout in educational and health contexts. Second, the instruments of this study were based on self-report which may be influenced by the social desirability of participants. Future studies could use multiple reporting from other educational agents and they could incorporate other qualitative instruments such as observational records and interviews to collect data. These strategies could be conducted with the teachers and academic leaders of the educational centers. It would also be interesting to include other psychoeducational variables such as self-efficacy, emotional intelligence, self-esteem and coping styles to evaluate their possible association with the burnout and engagement constructs. Finally, future investigations in this topic should be multicentric by including students’ data from several educative centers, degrees, different countries, and cultures.

### Implications for Practice

It is necessary to consider that demographic, occupational and academic factors of university occupational therapy (e.g., age, year level of enrolment, and hours directly spent working on occupational therapy studies, hours of indirect time spent working on occupational therapy studies, and hours spent per week engaged in self-care activities) were associated with burnout levels and educational engagement. The evidence derived from this information could generate a greater awareness in educators about the real emotional state of their students. Therefore, these factors could be relevant variables in the design of psychoeducational intervention programs in this population.

Base on the above findings, the information obtained can be also useful in an increasing number of situations and contexts for future educational diagnostic purposes as well as for psychoeducational orientation. This orientation could be focused in the promotion of wellbeing, positive personal resources, mental health, a higher performance of occupational therapy undergraduate students, and a decrease of levels of academic stress. Educators could make recommendations on the aspects of engagement that are best for students and to prevent exhaustion.

## Conclusion

The results indicate that a number of demographic and academic study factors were significantly associated with the three dimensions of burnout reported by undergraduate occupational therapy students. In addition, several demographic and academic factors were found to be significant predictors of the three components of educational engagement, those being vigor, dedication, and absorption. Being familiar with demographic and academic factors that are significant predictors in maintaining undergraduate occupational therapy students’ education engagement can provide valuable insights for educators in the university context. These conclusions provide important information for academic and fieldwork educators so that strategies can be put in place to manage chronic stress in undergraduate students.

## Data Availability Statement

The datasets generated for this study are available on request to the corresponding author.

## Ethics Statement

The studies involving human participants were reviewed and approved by the Monash University Human Research Ethics Committee approval was obtained on the April 09, 2015 (project number: 2015-6069-5898). This study met the requirements of the National Statement on Ethical Conduct in Human Research and was conducted in accordance with the Declaration of Helsinki 2013. The participants completed an individual informed consent to participate in the study. The patients/participants provided their written informed consent to participate in this study.

## Author Contributions

JP-M and TB conceived and designed the study, recruited the participants, and contributed to the manuscript writing and data analysis. FM-R contributed to the bibliographic review, manuscript writing, and data analysis. All authors revised the manuscript critically and approved the final version of the manuscript.

## Conflict of Interest

The authors declare that the research was conducted in the absence of any commercial or financial relationships that could be construed as a potential conflict of interest.

## Appendix 1

### Demographic, Occupational and Academic Questionnaire

The demographic, occupational and academic characteristics were elicited from participants using the following questions: (1) Which year level are you currently enrolled in? (First, Third, and Fourth year); (2) Are you currently enrolled in your occupational therapy studies (full-time, part-time or other); (3) Did you enter your occupational therapy education program directly from high school? (yes, no); (4) Did you have previous educational experience (e.g., other study at university, college or polytechnic) before enrolling in your current occupational therapy program? (yes, no); (5) How many hours of direct time (e.g., lectures, tutorials, practical skills classes, and laboratories, etc.) do you typically attend your occupational therapy education program each week? (hours); (6) How many hours of indirect time (e.g., reading, reviewing notes, preparing assignments, and listening to recorded lectures, etc.) do you typically spend working on and studying material related to your occupational therapy education program each week? (hours); (7) How many hours in a typical week do you work at a job (part time or full time) that you are paid for while attending your occupational therapy education program? (hours); and (8) How many hours in a typical week do you spend engaged in self-care activities (e.g., shopping, meal preparation, laundry, house cleaning, child care)? (hours).
